# Ultraviolet laser induced periodic surface structures positively influence osteogenic activity on titanium alloys

**DOI:** 10.3389/fbioe.2024.1462232

**Published:** 2024-10-28

**Authors:** Luiz Schweitzer, Janosch Schoon, Niklas Bläß, Katrin Huesker, Janine V. Neufend, Nikolai Siemens, Sander Bekeschus, Rabea Schlüter, Peter Schneider, Eckart Uhlmann, Georgi Wassilew, Frank Schulze

**Affiliations:** ^1^ Fraunhofer Institute for Production Systems and Design Technology, Berlin, Germany; ^2^ Center for Orthopaedics, Trauma Surgery and Rehabilitation Medicine, University Medicine Greifswald, Greifswald, Germany; ^3^ Endocrinology and Immunology Department, Institute for Medical Diagnostics, Berlin, Germany; ^4^ Department of Molecular Genetics and Infection Biology, University of Greifswald, Greifswald, Germany; ^5^ ZIK plasmatis, Leibniz Institute for Plasma Science and Technology (INP), Greifswald, Germany; ^6^ Clinic and Policlinic for Dermatology and Venerology, Rostock University Medical Center, Rostock, Germany; ^7^ Imaging Center of the Department of Biology, University of Greifswald, Greifswald, Germany; ^8^ Laser-Mikrotechnologie Dr. Kieburg, Berlin, Germany; ^9^ Institute for Machine Tools and Factory Management, Technische Universität Berlin, Berlin, Germany

**Keywords:** arthroplasty, biofilm, human osteoblasts, laser-induced periodic surface structures, cytokine release, surface topography

## Abstract

**Background/Objective:**

Endoprostheses might fail due to complications such as implant loosening or periprosthetic infections. The surface topography of implant materials is known to influence osseointegration and attachment of pathogenic bacteria. Laser-Induced Periodic Surface Structures (LIPSS) can improve the surface topography of orthopedic implant materials. In this preclinical *in vitro* study, laser pulses with a wavelength in the ultraviolet (UV) spectrum were applied for the generation of LIPSS to positively influence formation of extracellular matrix by primary human Osteoblasts (hOBs) and to reduce microbial biofilm formation *in vitro*.

**Methods:**

Laser machining was employed for generating UV-LIPSS on sample disks made of Ti6Al4V and Ti6Al7Nb alloys. Sample disks with polished surfaces were used as controls. Scanning electron microscopy was used for visualization of surface topography and adherent cells. Metal ion release and cellular metal levels were investigated by inductively coupled plasma mass spectrometry. Cell culture of hOBs on sample disks with and without UV-LIPSS surface treatments was performed. Cells were investigated for their viability, proliferation, osteogenic function and cytokine release. Biofilm formation was facilitated by seeding *Staphylococcus aureus* on sample disks and quantified by wheat germ agglutinin (WGA) staining.

**Results:**

UV-LIPSS modification results in topographies with a periodicity of 223 nm ≤ λ ≤ 278 nm. The release of metal ions was found increased for UV-LIPSS on Ti6Al4V and decreased for UV-LIPSS on Ti6Al7Nb, while cellular metal levels remain unaffected. Cellular adherence was decreased for hOBs on UV-LIPSS Ti6Al4V when compared to controls while proliferation rate was unaffected. Metabolic activity was lower on UV-LIPSS Ti6Al7Nb when compared to the control. Alkaline phosphatase activity was upregulated for hOBs grown on UV-LIPSS on both alloys. Less pro-inflammatory cytokines were released for cells grown on UV-LIPSS Ti6Al7Nb when compared to polished surfaces. WGA signals were significantly lower on UV-LIPSS Ti6Al7Nb indicating reduced formation of a *S. aureus* biofilm.

**Conclusion:**

Our results suggest that UV-LIPSS texturing of Ti6Al7Nb positively influence bone forming function and cytokine secretion profile of hOBs *in vitro*. In addition, our results indicate diminished biofilm formation on UV-LIPSS treated Ti6Al7Nb surfaces. These effects might prove beneficial in the context of long-term arthroplasty outcomes.

## 1 Introduction

Arthroplasty is a common procedure to restore mobility and quality of life in patients suffering from joint pathologies. Despite general success, a considerable number of joint implants fail due to loosening and periprosthetic joint infection. It is estimated that 635,000 hip and 1,280,000 knee replacements will be conducted in the United States alone in 2030 with 10% requiring revision due to failure (Sloan et al., 2018). To avoid implant loosening, the material of choice should be biocompatible and immunomodulatory. In addition, osseointegration should be promoted to form a stable interface between implant and surrounding bone tissue (Overmann et al., 2020). Since periprosthetic infection is one of the leading causes of implant failure, the attachment of pathogens and subsequent biofilm formation needs to be minimized (Quinn et al., 2020). Surface topography was identified early as a crucial factor for the successful integration of implants into bone tissue (Wennerberg and Albrektsson, 2009). Furthermore, the surface topography of a material influences the attachment of pathogenic bacteria thus reducing the formation of biofilms (Cheng et al., 2019). Titanium (Ti) and its alloys are widely used as implant materials in orthopedic surgery due to their favorable mechanical properties and good biocompatibility. The increased surface roughness of Ti implants has a positive effect on bone interface formation, osteoconduction and osteogenesis (Cooper et al., 2006). The Ti6Al4V alloy containing aluminum (Al) and vanadium (V) has been most commonly applied in the past and is still in use for many orthopedic implants, especially in knee arthroplasty. However, Ti6Al7Nb that utilizes niobium (Nb) instead of V has gradually replaced Ti6Al4V in total hip arthroplasties and is reported to exhibit comparable lower acute toxicity (Bozoglan and Dundar, 2021).

Laser-based modification of Ti surfaces offers unique advantages, such as minimization of impurities and the ability to create defined micro- and nanoscale structures (Schnell et al., 2019; Ferraris et al., 2024). Ultra-short pulse laser techniques offer distinct advantages, such as reduced surface contamination and minimized melting effects (Vorobyev and Guo, 2007). Among different laser-based methods for the creation of specific surface topographies, Laser-Induced Periodic Surface Structures (LIPSS) have garnered high interest. Unlike other techniques that create porous or crater-like structures, LIPSS create repetitive wave-like surface topographies that have found diverse applications ranging from altering surface wetting (Cunha et al., 2013) over tribological uses (Yasumaru et al., 2008), and cell culture applications (Wallat et al., 2012) to anti-reflective coatings (Granados et al., 2017). These structures typically emerge near the material’s ablation threshold under polarized laser exposure (Cunha et al., 2013). Generally, two kinds of LIPSS are recognized: Low Spatial Frequency LIPSS (LSFL) and High Spatial Frequency LIPSS (HSFL), differentiated by their spatial periodicity in relation to the laser wavelength λ used (Bonse et al., 2012). Ti and its alloys are amenable to LSFL LIPSS machining, leading to improved cellular adhesion, viability and bone matrix formation (Maalouf et al., 2022). In previous studies, the surfaces of Ti alloys have been textured by using wavelengths of λ = 532 nm (green radiation) and λ = 355 nm (ultraviolet (UV) radiation) achieving spatial periodicities of 404 nm ≤ λ ≤ 492 nm and 210 nm ≤ λ ≤ 274 nm, respectively on Ti6Al4V surfaces. The two surfaces were characterized for their topographies, physical properties and cytocompatibility and it was found that UV-LIPSS had favorable effects on mesenchymal stromal cells in terms of increased cell survival (Schweitzer et al., 2020). These studies concluded that UV-LIPSS might be favorable for the surface modification of orthopedic implants to enhance bone ingrowth and should be investigated further. For implant ingrowth, bone tissue needs to undergo significant turnover facilitated by the coordinated resorption and formation of bone-like extracellular matrix. This process, termed remodeling, is conducted by bone-forming osteoblasts (OBs) and bone-resorbing osteoclasts (OCs) (Al-Bari and Al, 2020). The coordinated interaction of these cell types is mediated by cytokines that are secreted into the extracellular space. A disproportion between bone formation and resorption can lead to a reduction in bone mass (Hartmann et al., 2017). For the formation of new tissue, OBs secrete collagen type I that is subsequently mineralized (Ponzetti and Rucci, 2021). To facilitate successful implant ingrowth, it is thus of paramount importance that the implant material allows for proper adhesion and function of OBs while cell viability should not be influenced negatively. In addition, implant materials should be modified to minimize pro-inflammatory reactions that are often accompanied by an increase in OC activity. It is well described that the migration of macrophages to peri-implant tissues can lead to accelerated osteolysis and subsequent aseptic implant loosening (Huzum et al., 2021). The recruitment and activity of different immune cells are modulated by cytokines secreted by tissue-resident cells such as OBs (Guder et al., 2020). Thus, the effect of novel materials on OB viability, function and cytokine secretion should be investigated thoroughly as these parameters might influence the long-term stability of the respective arthroplasty implants.

In this work, it is hypothesized that the generation of UV-LIPSS on Ti6Al4V and Ti6Al7Nb will positively influence osteogenic function of OBs while reducing attachment of bacterial biofilms. To verify this hypothesis, a testing strategy was employed that utilizes primary human OBs (hOBs). The cells were seeded onto the different surfaces and investigated for their viability and osteogenic function while their cytokine secreting profile was studied to gain insights into possible immunogenic interactions. In addition, the influence of UV-LIPSS on biofilm formation of *Staphylococcus aureus,* a clinically relevant pathogen, was investigated. In summary, this study aimed to (a) optimize process parameters to generate UV-LIPSS on Ti6Al7Nb surfaces, (b) to thoroughly characterize the influence of UV-LIPSS on hOB functions *in vitro* that are vital for proper implant ingrowth and positive arthroplasty outcome and (c) to investigate how bacterial adhesion and subsequent biofilm formation is affected by UV-LIPSS.

## 2 Materials and methods

### 2.1 Sample material

Ti6Al7Nb and Ti6Al4V ELI raw material (High Tech Alloys Sonderwerkstoffe GmbH, Wuppertal, Germany) were used. These materials follow the norm ISO 5832-3 and ISO 5832-11 regarding their chemical composition as shown in Table 1. The samples were manufactured as disks, with a sample diameter d = 10.0 mm, sample height h = 2.0 mm, and sample phase angle α = 0.25 × 45.0. The samples were manufactured with a lathe (Deco 13; Tornos, Moutier, Switzerland) applying a specialized cutting tool (VBMT 110302-kf; Sandvik Coromant, Sandviken, Sweden). All sample disks were electropolished as a finishing process to remove debris and provide lower surface roughness. A electrolyte that is specific for titanium alloy dental implants (ElpoLux TI-Med; ElpoChem AG, Volketswil, Switzerland) was applied. Samples were separately treated according to the electrolyte manufacturer instructions and conditions at room temperature (T = 20 °C) with a polishing time of t_p_ = 5.0 min. The polished surfaces without further modifications (POL) were employed as reference within the *in vitro* experiments.

**TABLE 1:** Nominal chemical composition of the Ti-alloys.

Composition of Ti6Al4V alloy							
Element	Al	V	Fe	O	C	N	Ti
Weight fraction [%]	6.11	3.93	0.12	0.11	0.01	0.01	bal

Composition of the Ti6Al7Nb alloy										
Element	Nb	Al	O	Fe	C	N	V	H	Ta	Ti
Weight fraction [%]	6.97	6.11	0.17	0.14	0.006	0.005	0.004	0.003	0.001	bal

Abbreviation: bal, balance.

### 2.2 Laser-based surface modification

For generating the UV-LIPSS on polished sample disks, a laser machine tool (LMBS Tricolore; Laser Mikrotechnologie Dr. Kieburg, Berlin, Germany) was applied. It applied an ultrashort pulsed laser source (Talisker-Three; Coherent, Santa Clara, CA, United States) emitting UV radiation with a wavelength of λ = 355 nm. The source delivers a pulse duration τ = 10 ps and a maximum repetition rate f = 500 kHz. It has an average beam power *p* = 3.0 W and a laser beam diameter D = 12.0 μm at the focusing position. The surface structures manufactured in the present work are LSFLs produced by a repetition rate f = 200 kHz. For the manufacturing of UV-LIPSS, the technique described by Oliveira et al. was applied (Schweitzer et al., 2020; Oliveira et al., 2009). Thereby, the laser average fluence required for inducing the formation of the nanotextures was estimated. The texturing of the entire surface was realized with a scanner device (excelliSCAN 20; SCANLAB, Puchheim, Germany). The laser surface texturing of each sample was performed without the displacement of the machine axis for higher precision and surface quality. The laser beam was applied perpendicular to the sample and focused on the surface. Spatial periodicity was measured using a Atomic Force Microscope (AFM) (N8 NEOS; Brucker Corp., Billerica, United States).

### 2.3 Sample disk treatment prior experimentation

Polished and UV-LIPSS treated sample disks were further processed by means of cleaning and sterilization to remove residual particles and impurities from prior manufacturing and to allow for their use in cell culture. A three-step cleaning protocol was used for this purpose. The surfaces were cleaned in an ultrasonic bath (Elmasonic S10; Elma, Singen, Germany) at 80,000 Hz for 5 min in isopropyl alcohol (1157-5L; Th. Geyer, Renningen, Germany), acetone (8002 1L; Th. Geyer, Renningen, Germany), and phosphate-buffered saline (PBS; PAN-Biotech, Aidenbach, Germany). In a fourth step, the samples for surface characterization were cleaned with distilled water at 80,000 Hz for 5 min. The PBS solution removed residual alcohol that could affect the *in vitro* experiments.

### 2.4 Scanning electron microscopy

The surface morphology characterization of the samples was realized by scanning electron microscopy (SEM) (LEO 1455 VP; Carl Zeiss Microscopy, Jena, Germany). SEM analysis was conducted after *in vitro* experiments to characterize cellular adhesion on the sample surface. All samples have been coated with gold prior SEM analysis to ensure conductivity of their surface. The samples were treated using a sputter coater (Cressington 108 auto; TESCAN, Dortmund, Germany) with a coating time t_a_ = 0.08 h in an argon atmosphere with pressure *p* = 0.05 MPa.

### 2.5 Cell isolation and expansion

This study was approved by the Ethics Committee of the University Medicine Greifswald (Germany; ethical approval code: BB 178/20). All cell donors provided written informed consent. hOBs from n = 6 donors were isolated by outgrowth from cancellous bone and subsequent adherence to polystyrene tissue culture plastic (TCP). To remove the hematopoietic cells from harvested cancellous bone, red marrow was washed out with phosphate-buffered saline (PBS; PAN-Biotech, Aidenbach, Germany) until the remaining tissue appeared bright white. The bone fragments were then cut into small pieces with a sterile scalpel, and hOBs were isolated by incubation in TCP flasks under standard cell culture conditions (5% CO_2_, 37°C, 95% humidity). For the purification and subsequent expansion, hOBs were cultured in expansion media (EM) containing low glucose Dulbecco’s modified Eagle’s Medium (DMEM; PAN-Biotech, Aidenbach, Germany) supplemented with 10% fetal bovine serum (FBS superior; Sigma-Aldrich, St. Louis, MO, United States), 5 mM L-alanyl-L-glutamine (GlutaMAX; Thermo Fisher Scientific, Waltham, MA, United States), 100 U/mL penicillin and 100 μg/mL streptomycin (Penicillin-Streptomycin; Thermo Fisher Scientific, Waltham, MA, United States). Cells were passaged when 90% confluency was reached and cryopreserved at passage two. Cells were thawed and expanded until passage three prior to further use for the individual experiments as required. The different sample disks had the correct dimensions to be press fitted into the wells of a 48-well plate and their planar positioning was achieved using a retractable 3D-printed tool. Subsequently, the surfaces were covered with 200 µL of EM and incubated for 12 h under standard cell culture conditions (5% CO_2_, 37°C, 95% humidity) to ensure the formation of a protein layer for better cell adherence. For experiments regarding cell viability and function, the hOBs were seeded onto the sample disks and kept for 24 h under standard cell culture conditions to allow for cell adherence before they were cultivated further.

### 2.6 Metal ion release

Human osteoblasts from one donor were used and 12 × 10^3^ cells/wells were seeded on the respective disks (n = 10 for each surface treatment) in technical replicates. Tissue culture plastic (TCP) served as a control. All experiments were carried out on 48-well plates. Cells were grown until day 8 and sampling of cell culture media was performed for subsequent investigation of metal levels. The culture supernatants from two wells of a group were pooled consecutively (400 µL), collected in a cryotube, and stored at 4°C. To create a cell lysate, the cells were detached from the surface by incubation with trypsin (Trypsin 0.05%, EDTA 0.02%, w/o: Ca/MG; PAN-Biotech, Aidenbach, Germany). Subsequently, the cell suspension from two wells of the same surface was pooled. Each sample was fixed overnight at 4°C using a 4% formaldehyde solution. The cells were resuspended in 100 µL of PBS. From this volume, 50 µL were mixed with another 50 µL of lysis buffer (RLT-Plus; Quiagen, Venlo, Netherlands). The volume was adjusted to 1 mL with 900 µL of PBS. The culture supernatant and the cell lysate were diluted in 1% HNO_3_ (for the analysis of Ti, V, Nb) or 1% HCl (for the analysis of Al) and then measured using inductively coupled plasma mass spectrometry (ICP-MS) (ICapQ; Thermo Fisher GmbH, Bremen, Germany) in collision-reaction mode, normalized to internal standards (74Ge and 187Re). The precision of the measurement was verified using certified control material (Lyphochek Urine L1 and L2; Bio-Rad Laboratories GmbH, Munich, Germany).

### 2.7 Cell viability and proliferation

To investigate the cellular metabolic activity and rate of cell death, 4.8 × 10^3^ cells/well were seeded on the respective disks in quadruplicates. Cells were grown for 14 days and sampling of cell culture media was performed at day 0 (24 h after seeding), 4, 7, 11 and 14 for subsequent investigation of cell death. On the same days, cellular metabolic activity was determined using a resazurin-based assay (PrestoBlue; Invitrogen, Waltham, MA, United States) according to the manufacturer’s manual. Relative cell numbers were determined by quantification of DNA content using a cell proliferation assay (CyQUANT; Thermo Fisher Scientific, Waltham, MA, United States) according to the manufacturer’s protocol. The samples from cell culture media acquired on day 0 (24 h after seeding), 4, 7, 11, and 14 were used to determine levels of lactate dehydrogenase (LDH) as a marker for relative cell death. For these experiments, a commercial kit (LDH-Cytox Assay Kit; BioLegend, Amsterdam, Netherlands) was used according to the manufacturer’s instructions. Cells from one donor were seeded out on disks in a 48-well tissue culture plate in triplicates with a density of 9 × 10^3^ cells/well. After incubating 24 h under standard cell culture conditions, viability was determined using a live/dead staining kit (LIVE/DEAD Cell Imaging Kit; Invitrogen, MA, Waltham, United States) following the manufacturer’s instructions. Investigation of the stained cells and acquisition of representative images was performed using a fluorescence microscope (Evos FL Cell Imaging System; Invitrogen, Waltham, MA, United States).

### 2.8 Osteogenic function

For investigating hOB function, cells from one donor were stimulated with osteogenic medium (OM) and subsequently analyzed for their alkaline phosphatase (ALP) activity. Cells were seeded onto the respective disks in 48-well tissue culture plates at a density of 9 × 10^3^ cells/well. After 24 h adherence time, cells were either cultured in OM or EM in six technical replicates for each condition. The OM contained basic EM supplemented with 100 nM Dexamethasone, 50 µM ascorbic acid and 10 mM β-glycerol phosphate (all supplements from Sigma-Aldrich, MO, United States). After 10 days of total incubation time, the ALP activity was determined as described elsewhere (Krause et al., 2011). In brief, cells were washed using 400 μL of prewarmed PBS followed by 200 μL AP-Buffer (100 mM NaCl, 100 mM Tris, 1 mM MgCl2; pH 9.0). After this washing step, a mixture of 100 µL AP-buffer and 100 µL p-nitrophenyl phosphate (pNPP; Sigma-Aldrich, MO, United States) were added per well, followed by 10 min incubation at standard cell culture conditions. After incubation, the reaction was stopped by adding 200 µL of 1M NaOH, followed by transferring 2 × 100 µL of the mixture into the wells of a 96-well tissue culture plate for subsequent absorbance measurements at 405 nm on a multiplate reader (M200; Tecan, Männedorf, Switzerland).

### 2.9 Cytokine release

Cytokine release was investigated using the cell culture supernatant samples acquired in the proliferation experiments on day 7. The samples from all six technical replicates were pooled and stored at −80°C until further processing. A microbead-based multianalyte-sandwich-ELISA (LEGENDplex Custom Human 12-plex Panel; BioLegend, Amsterdam, Netherlands) was used to determine the concentration of the following 12 cytokines in four technical replicates: tissue inhibitor of metalloproteinase 2 (TIMP-2), osteoprotegerin (OPG), hepatocyte growth factor (HGF), stromal cell-derived factor 1 (SDF-1), stem cell factor (SCF), transforming growth factor beta 1 (TGF-β1), vascular endothelial growth factor A (VEGF-A), interleukin 6 (IL-6), interleukin 8 (IL-8), monocyte colony stimulating factor (M-CSF), monocyte chemoattractant protein 1 (MCP-1) and interleukin receptor antagonist protein 1 (IL-1RA). Cytokines were investigated due to their role in inflammatory processes such as the recruitment or activation of immune cells (M-CSF, MCP-1, SDF-1), the inflammatory response of these cells (Il-6, IL-8, IL-1RA) and the expression of growth factors relevant for immune cells (HGF, SCF, TGF-ß1). Samples were analyzed using a flow cytometer (CytoFLEX LX; Beckman-Coulter, Brea, CA, United States). Concentrations were determined using dedicated software (LegendPLEX 8.0; Vigene Tech, Carlisle, MA, United States).

### 2.10 Investigations of cell adherence by scanning electron microscopy

Cells were grown on sample disks for 14 days and subsequently fixated by incubation with 4% formaldehyde for 10 min at RT. After fixation, cells were washed three times in PBS for 5 min each time, and then dehydrated in a graded series of aqueous ethanol solutions (10%, 30%, 50%, 70%, 90%, 100%) on ice for 15 min each step. The samples were then allowed to reach RT before the ethanol was replaced with fresh 100% ethanol at RT for 10 min. Subsequently, samples were critical point-dried with liquid CO_2_. Finally, samples were mounted on aluminum stubs, sputtered with gold/palladium (Au/Pd) and examined using SEM (EVO LS10; Carl Zeiss Microscopy Deutschland GmbH, Oberkochen, Germany). All micrographs were edited by using Adobe Photoshop CS6.

### 2.11 Investigations on biofilm formation

*Staphylococcus aureus* (*S. aureus*) strain INFECT6005/ST20140625 (Baude et al., 2019) was grown overnight as described elsewhere (Mairpady Shambat et al., 2016). To investigate biofilm formation, *S. aureus* was seeded on polished and UV-LIPSS surfaces of Ti6Al7Nb samples. Biofilm assays were performed as previously described (Lembke et al., 2006) using 48-well plates and n = 6 polished and n = 6 UV-LIPSS Ti6Al7Nb sample disks. Briefly, overnight cultures of strain INFECT6005/ST20140625 were pelleted, washed three times with PBS, and suspended to a final OD [600 nm] of 0.07 in brain-heat-infusion media supplemented with 2% (w/v) glucose (both Carl Roth, Karlsruhe, Germany). The bacterial suspensions were seeded into the well plate and incubated at 37°C and 5% CO_2_. After 48h, biofilms were washed twice with PBS, fixed with 2% (v/v) paraformaldehyde for 10 min, washed twice with PBS, and stained with wheat germ agglutinin (WGA) conjugated with Alexa Fluor 488 (final concentration: 10 μg×ml-1; Invitrogen, Waltham, MA, United States) for 10 min. Samples were mounted with mounting medium containing DAPI (Fluoroshield; Abcam, Cambridge, United Kingdom). Images of biofilm grown on the respective parts were acquired using an inverted fluorescence microscope (Evos FL Cell Imaging System; Invitrogen, MA, United States) at ×10 magnification. Images were analyzed using the Fiji software package (Schindelin et al., 2012). To take biofilm heterogeneity into account, three different images were acquired per sample for each surface treatment. The images were postprocessed for their brightness and contrast using the same parameters on each image. Images were then binarized using a fixed threshold that was applied for every image to determine the mean grey value of the respective staining. The values of WGA staining were then referenced to the values of the DAPI staining to normalize the biofilm signal.

### 2.12 Statistical analysis

According to the research question addressed and the corresponding hypothesis, the differences between polished and UV-LIPSS treated surfaces were investigated and thus the aim of statistical analysis. The direct comparison between Ti6Al4V and Ti6Al7Nb was not the aim of the current study and was thus not reflected in statistical testing. Therefore, only pairwise testing of polished and UV-LIPSS treated surfaces was performed for each of the respective alloy and a one-way ANOVA for all tests performed at different time points. All data was tested for normal distribution and presented as mean with standard deviation. In the experiments on quantification of viability, proliferation capacity, quantification of LDH release, and induction of osteogenic matrix mineralization an ordinary one-way ANOVA with post-hoc Bonferroni correction was performed. The statistical analyses in the experiments on metal release and quantification of soluble cytokines were conducted using an unpaired t-test. The biofilm formation data was found to be non-normally distributed and thus tested using a non-parametric Mann-Whitney Test. The exact number of technical and/or biological replicates can be found in the respective figure texts. Results with *p* ≤ 0.05 were considered statistically significant. For data analysis and visualization, GraphPad Prism Version 9.5.1 (GraphPad Software; La Jolla, CA, United States) was used.

## 3 Results

### 3.1 Determination of optimum process parameters

**FIGURE 1:**
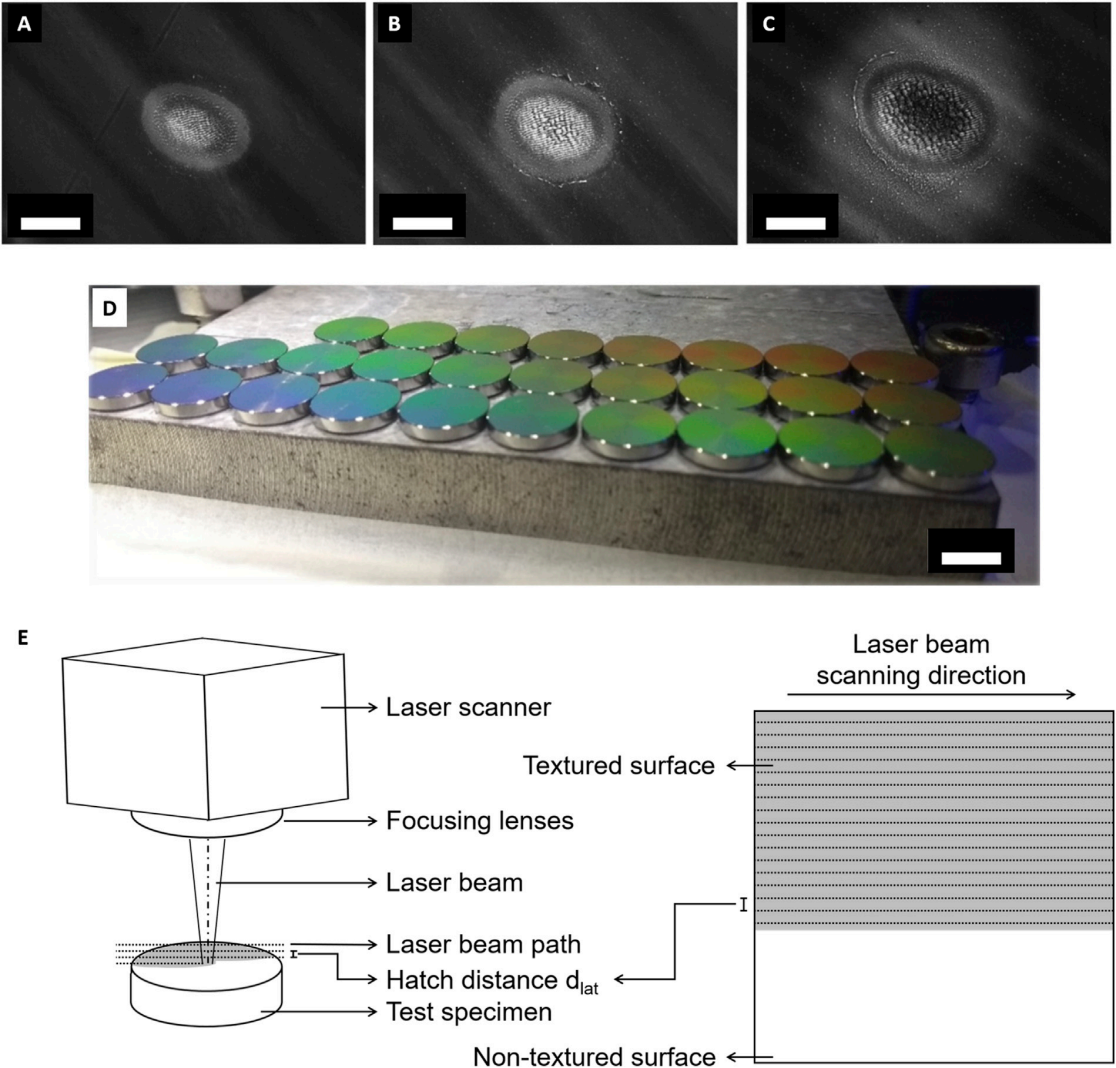
Laser-induced surface texturing. Ti6Al4V samples were evaluated in regard to the surface morphologies according to the energy E pulse applied on the surface. **(A)** The UV-LIPSS formation started in the center at E_p_ = 0.050 µJ. **(B)** The ripples started to form along the crater by applying E_p_ = 0.070 µJ. **(C)** The increase of the pulse energy to E_p_ = 0.090 µJ led to the formation of microcolumns in the center, while the ripples are still recognized on the periphery. They form as a result of the ripples collapse, after its first formation of parallel structures on the surface. **(D)** Samples after laser-induced surface texturing. **(E)** Shown is the experimental setup for the surface texturing. (scalebar in **(A–C)** indicate b = 5 μm, scalebar in **(D)** indicates b = 10 mm).

In this work, 25 ≤ N ≤ 200 laser pulses were implied while the used pulse energy E_p_ range of 0.050 µJ ≤ E_p_ ≤ 0.110 µJ led to the formation of different surface textures (Figure 1). Figures 1A–C show the results of single craters formed by focused laser radiation applied perpendicular to the surface. The micrographs were acquired using an SEM. At E_p_ = 0.050 µJ, the UV-LIPSS formation on Ti6Al4V started in the disks’ center (Figure 1A). The formation of ripples along the crater was achieved at E_p_ = 0.070 µJ (Figure 1B). The ripples show a spatial periodicity in the range of in 230 nm ≤ Λ ≤ 265 nm, as investigated by AFM. By increasing the pulse energy to E_p_ = 0.090 µJ, microcolumns appeared in the center of the crater, while the ripples were still recognized on the periphery (Figure 1C). At E_p_ = 0.090 µJ, both UV-LIPSS and microcolumns grew further to the periphery of the crater. In its center, the applied energy E induced the collapsing of the microcolumns, forming a flat molten region. By further increase of the pulse energy E_p_, this behavior led to a collapse of all textures, resulting in laser ablation. The same behavior was observed on the Ti6Al7Nb samples. In this case, slight changes in the pulse energy E_p_ led to similar formations on the single craters. The UV-LIPSS formation started at E_p_ = 0.074 µJ. The collapse of the UV-LIPSS structures occurred after reaching E_p_ = 0.084 µJ (data not shown). For the processing of the entire sample surface, lateral increment between adjacent tracks was required. The hatch distance determined the overlapping of tracks and thereby the overlap rate required for the formation of UV-LIPSS over the entire surface. Another processing parameter that directly influenced the texture formation was the scanning speed applied. The manufacturing of the laser textures to Ti6Al4V required the application of an average fluence of F = 0.047 J/cm^2^ and a scanning speed v = 112 mm/s. The hatch distance applied between sequential laser tracks resulted in the lateral overlap of 87% (Figure 1D). For Ti6Al7Nb, the processing parameters corresponded to an average fluence of F = 0.052 J/cm^2^ and a scanning speed v = 107 mm/s. The UV-LIPSS formation was achieved with lateral overlap rate of 91%. A summary of all laser processing parameters is given in Table 2.

**TABLE 2:** Laser parameters for UV-LIPSS.

	Ep [µJ]	F [J/cm^2^]	V [mm/s]	Lateral overlap [%]
Ti6Al4V	0.090	0.047	112	87
Ti6Al7Nb	0.074	0.052	107	91

### 3.2 Cell adherence on the surface

**FIGURE 2:**
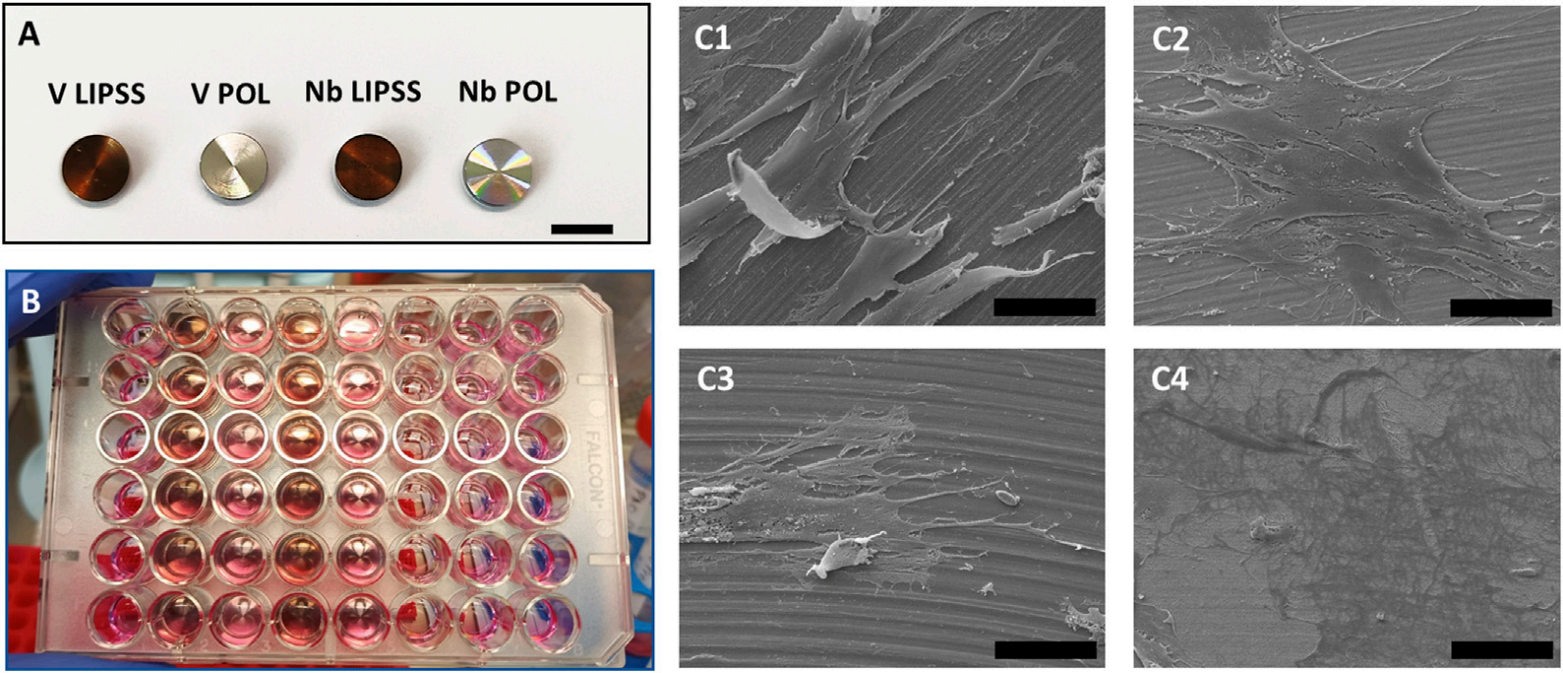
General setup for hOB cell seeding and subsequent culture. **(A)** Ti6Al4V or Ti6Al7Nb disks with polished (POL) or modified (LIPSS) surfaces (scale bar indicates b = 10 mm). **(B)** The respective disks where inserted into a 48-well tissue culture plate for cell seeding. **(C)** Shown are representative SEM images that confirm the attachment of hOBs to V POL (C1), V LIPSS (C2), Nb POL (C3) and Nb LIPSS (C4) after cell seeding (scale bar indicates b = 50 µm). Abbreviations: V POL, polished Ti6Al4V disks; V LIPPS, UV-LIPSS textured Ti6Al4V disks; Nb POL, polished Ti6Al7Nb disks; Nb LIPPS, UV-LIPSS textured Ti6Al7Nb disks.

Laser-textured and polished disks (Figure 2A) were fitted in a 48-well plate for subsequent cell seeding (Figure 2B), and cell adherence was examined by scanning electron microscopy. On the polished surfaces (Figures 2C1, C3), and the textured surfaces (Figures 2C2, C4), the cells are evenly spread confirming their adhesion to both surfaces qualitatively.

### 3.3 Metal ion release

**FIGURE 3:**
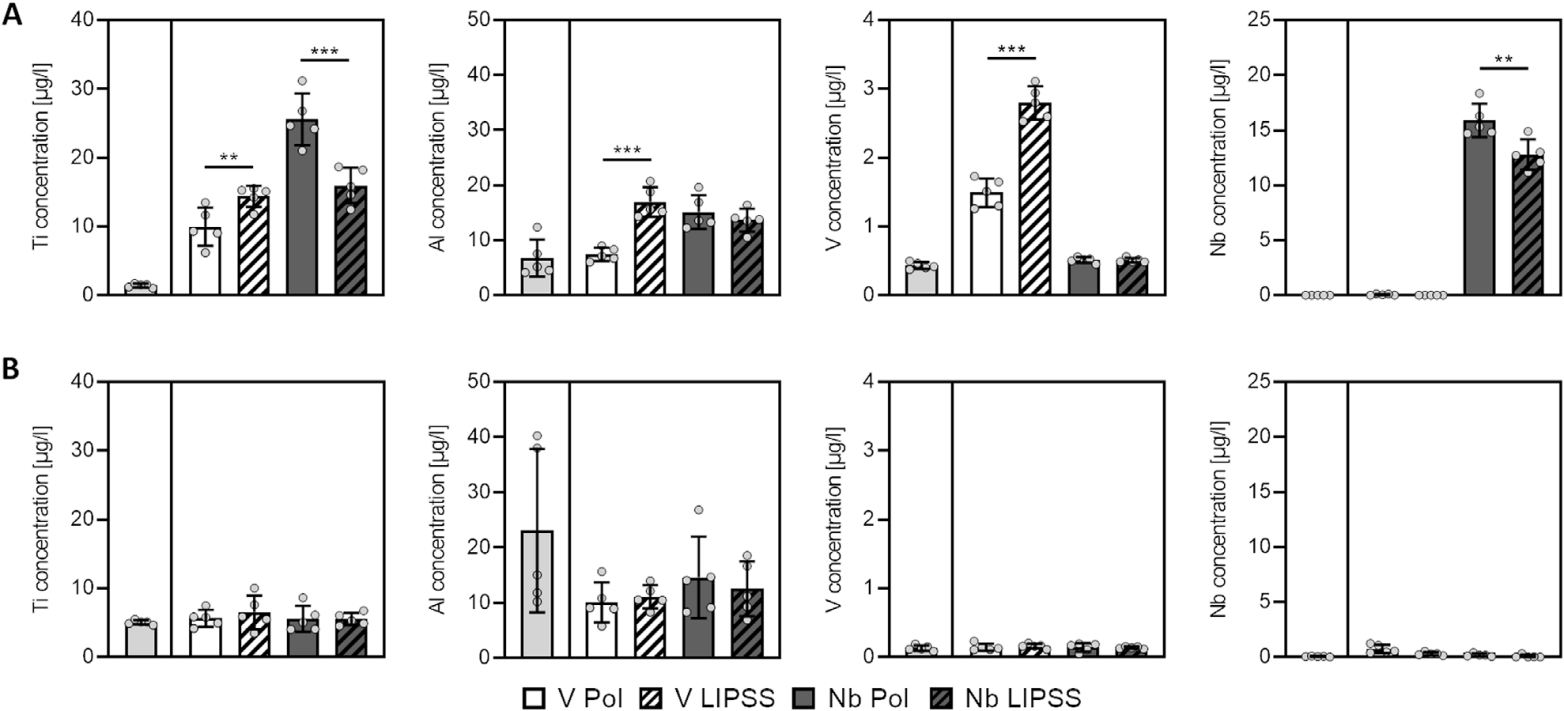
Dissolution of alloy metal ions depends on surface treatment and used alloy while cellular metal ion levels are not affected. Shown are metal concentrations in **(A)** cell culture media and **(B)** cell lysates after 7 days. (Student’s t-test, * indicates *p* ≤ 0.05, ** indicates *p* ≤ 0.010, *** indicates *p* ≤ 0.001). Abbreviations: TCP, tissue culture plastic controls; V POL, polished Ti6Al4V disks; V LIPPS, UV-LIPSS textured Ti6Al4V disks; Nb POL, polished Ti6Al7Nb disks; Nb LIPPS, UV-LIPSS textured Ti6Al7Nb disks.

The release of metal ions from alloys can lead to cytotoxicity at the peri-implant interface. Therefore, the release of metal ions under standard cell culture conditions was tested for the two alloys and their surface treatments employed in this work. The release of Ti, Al, and V was significantly increased from UV-LIPSS-treated disks made of Ti6Al4V (V LIPSS) when compared to their polished counterparts (V POL) (Figure 3A). In contrast, significantly less Ti and Nb were released from disks made of the Ti6Al7Nb alloy with UV-LIPSS surfaces (Nb LIPSS) when compared to the polished disks (Nb POL) (Figure 3A). The cellular uptake of metal ions was not affected by the surface treatment or alloy used (Figure 3B). Taken together, the effect of surface treatments on metal ion release seems to be a function of the alloy employed while cellular metal ion uptake is not affected.

### 3.4 Viability and proliferation of human osteoblasts

**FIGURE 4:**
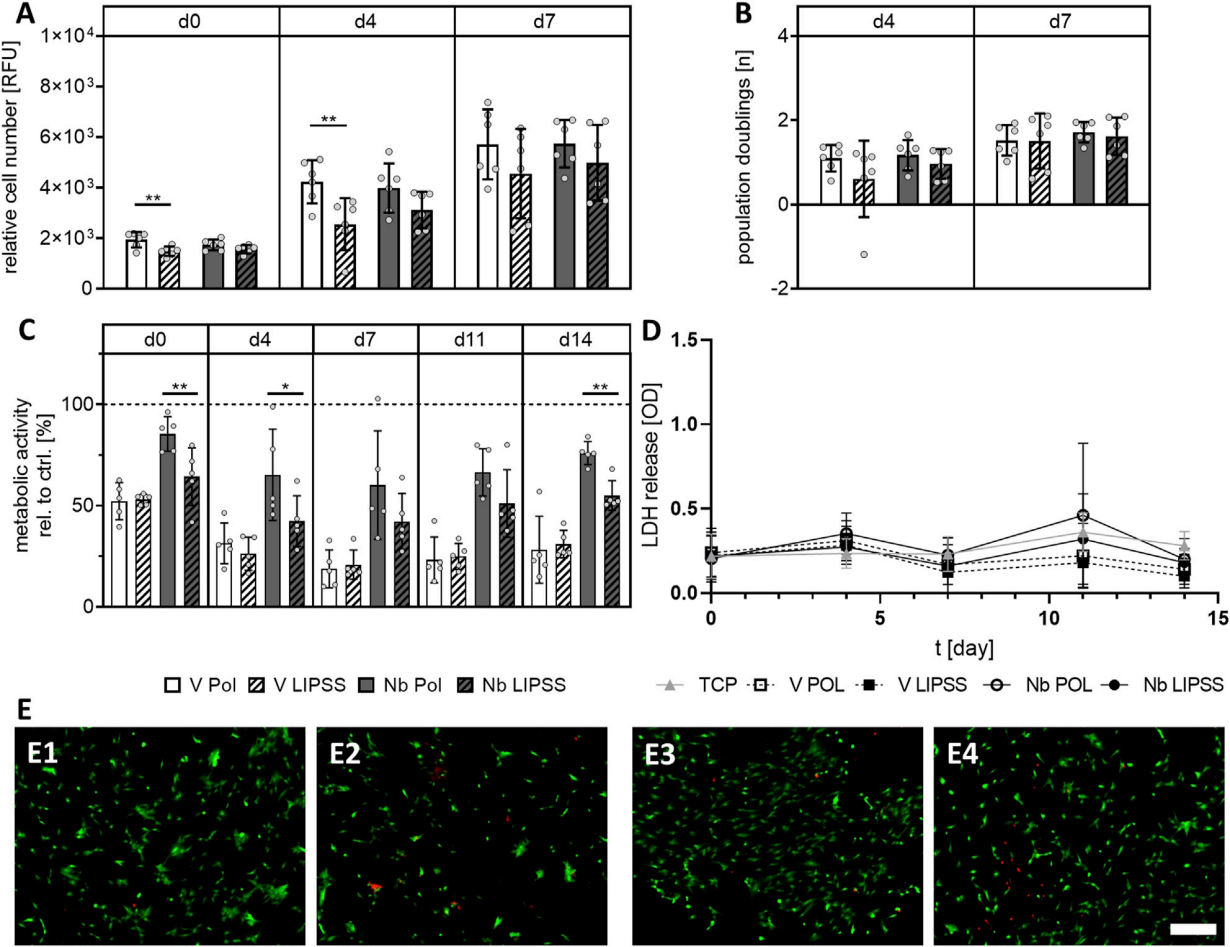
Viability and Proliferation of hOBs grown on different alloys that were either polished or LIPSS treated. **(A)** Shown are relative cell numbers at day 0 (24 h after seeding), day 4 and day 7. **(B)** The growth rate of hOBs on the different surfaces shown as population doubling rates. **(C)** Metabolic activity of hOBs over time. **(D)** Relative cell death indicated by LDH release. **(E)** Representative images of a live-dead staining on the different surfaces: V POL (E1), V LIPSS (E2), Nb POL (E3) and Nb LIPSS (E4). (scale bar indicates b = 500 μm, one-way ANOVA with Bonferroni’s posthoc test for multiple comparisons, * indicates *p* ≤ 0.05, ** indicates *p* ≤ 0.01, n = 6 biological replicates). Abbreviations: Nb POL, polished Ti6Al7Nb disks; Nb LIPPS, UV-LIPSS textured Ti6Al7Nb disks; V POL, polished Ti6Al4V disks; V LIPPS, UV-LIPSS textured Ti6Al4V disks; TCP, tissue culture plastic.

After quantification of metal ion release, viability and proliferation of hOBs grown on the different sample disks were determined. Relative cell numbers were found to be significantly decreased at days 0 and 4 for cells grown on V LIPSS when compared to V POL, while the actual cell proliferation rate was not affected as demonstrated by similar population doublings (Figures 4A, B). The different surface treatments had no effect on relative cell number or proliferation rate when Ti6Al7Nb was used. For the Ti6Al4V alloy, the surface treatment employed did not affect metabolic activity. In contrast, the metabolic activity of hOBs was significantly lower when grown on UV-LIPSS-treated surfaces for sample disks made of the Ti6Al7Nb alloy (Figure 4C). The cell death rate, indicated by LDH release, for hOBs did not exhibit significant differences between LIPSS treated surfaces and their polished counterparts (Figures 4D, E). Reduced cell numbers found on UV-LIPSS-treated disks made of Ti6Al4V were confirmed by the observation that fewer cells attach to V LIPSS when compared to the V POL alloy, while cells seeded on Nb POL and Nb LIPSS seem to be equal in terms of distribution and number (Figure 4E). In summary, UV-LIPSS surface treatment resulted in different effects on cell viability and adhesion depending on the alloy used. For Ti6Al4V, initial cell numbers were found to be reduced on UV-LIPSS surfaces indicating reduced adhesion. Cells grown on Ti6Al7Nb with UV-LIPSS exhibited a significant lower metabolic activity when compared to their polished counterparts.

### 3.5 Osteogenic function and cytokine release

**FIGURE 5:**
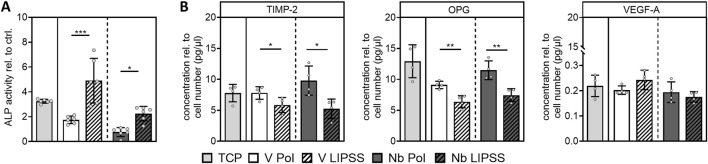
Osteogenic function of hOBs. **(A)** Shown is the ALP activity of hOBs after osteogenic stimulus relative to the non-stimulated control **(B)** Release of TIMP-2, OPG and VEGF of hOBs grown on the respective surfaces. (Student’s t-test, n ≥ 3 technical replicates, n = 1 cell donor, * indicates *p* ≤ 0.05, ** indicates *p* ≤ 0.01, *** indicates *p* ≤ 0.001) Abbreviations: Nb POL, polished Ti6Al7Nb disks; Nb LIPPS, UV-LIPSS textured Ti6Al7Nb disks; V POL, polished Ti6Al4V disks; V LIPPS, UV-LIPSS textured Ti6Al4V disks; TCP, tissue culture plastic.

**FIGURE 6:**
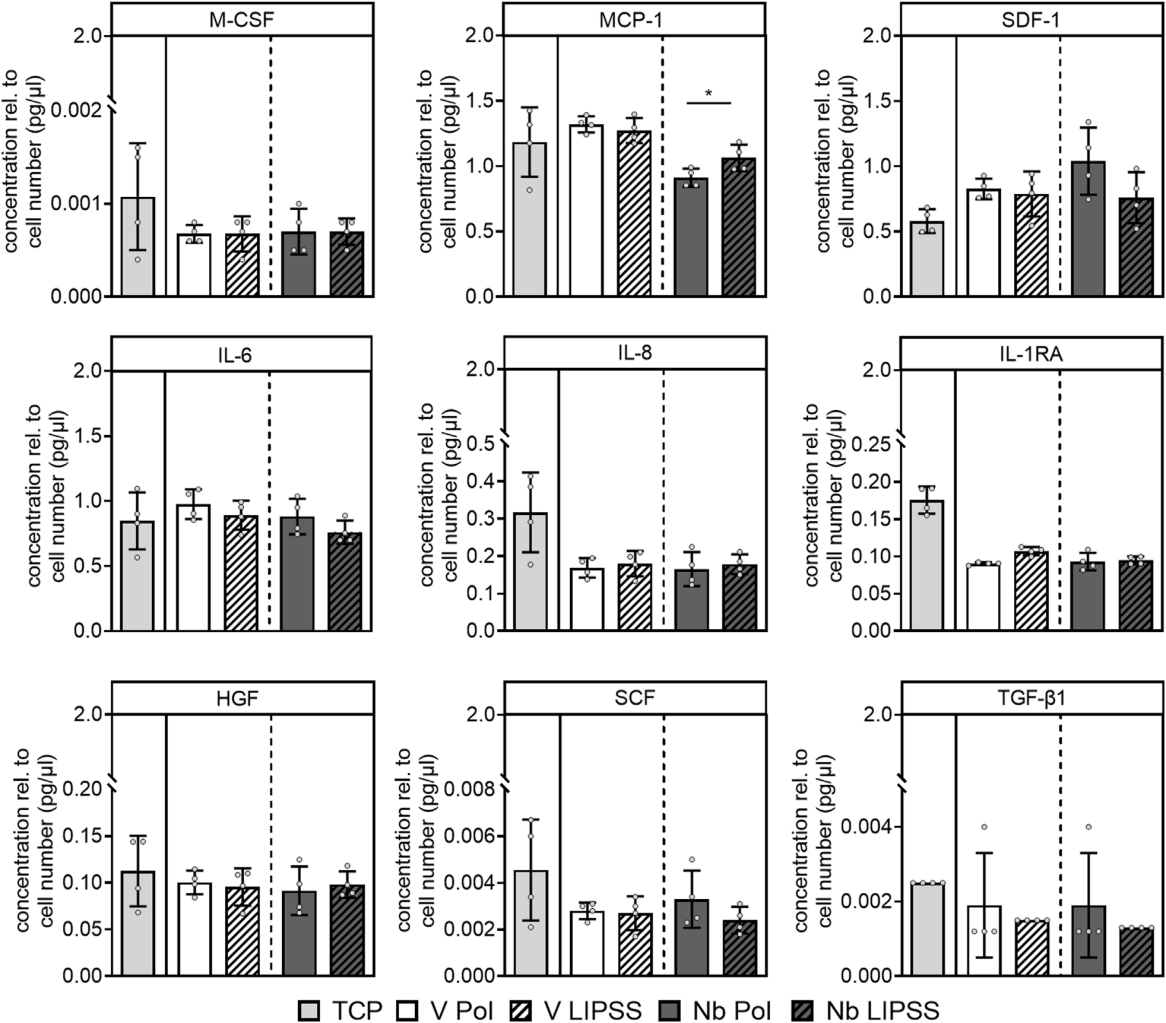
Baseline inflammatory cytokine expression profile of hOBs. The release of different inflammatory cytokines following culture of hOBs on the respective materials and surfaces. (student’s t-test, n ≥ 3 technical replicates, n = 1 cell donor, (Student’s t-test, n ≥ 3 technical replicates, n = 1 cell donor, * indicates *p* ≤ 0.05, ** indicates *p* ≤ 0.01, *** indicates *p* ≤ 0.001). Abbreviations: Nb POL, polished Ti6Al7Nb disks; Nb LIPPS, UV-LIPSS textured Ti6Al7Nb disks; V POL, polished Ti6Al4V disks; V LIPPS, UV-LIPSS textured Ti6Al4V disks; TCP, tissue culture plastic.

Functional investigations regarding the hOBs osteogenic activity and inflammatory response were conducted. Both factors play a crucial role in ingrowth and survival of implants. It was found that ALP activity of cells grown on UV-LIPSS-treated disks is significantly increased, independent of the alloy used (Figure 5A). Next, the release of the remodeling marker proteins TIMP-2, OPG and VEGF-A by cells grown on different surfaces was investigated. OPG and TIMP-2 were employed as markers for bone degradation in the course of bone matrix remodeling. Here, the UV-LIPSS treatment did result in significantly less release of TIMP-2 and OPG on both alloys (Figure 5B). Expression levels of the pro-angiogenic factor VEGF-A were unaffected by the surface treatment or alloy employed (Figure 5B). Aseptic inflammation can be the cause for implant loosening. It was therefore investigated if cytokine release is altered by the respective materials and surface treatments. Under standard cell culture conditions using EM, no significant effects on cytokine expression were found except for a significant increase in release of MCP-1 by hOBs grown on UV-LIPSS textured Ti6Al7Nb when compared to cells cultivated on the polished counterpart (Figure 6). In summary, matrix mineralization seems to be upregulated in cells grown on UV-LIPSS treated disks as indicated by the significant increase in ALP activity. In addition, the release of factors that govern bone resorption seems to be reduced for cells grown on UV-LIPSS surfaces, as indicated by lower levels of TIMP-2 and OPG found in the respective cell culture supernatants.

### 3.6 The effect of osteogenic stimulation on cytokine and growth factor release

**FIGURE 7:**
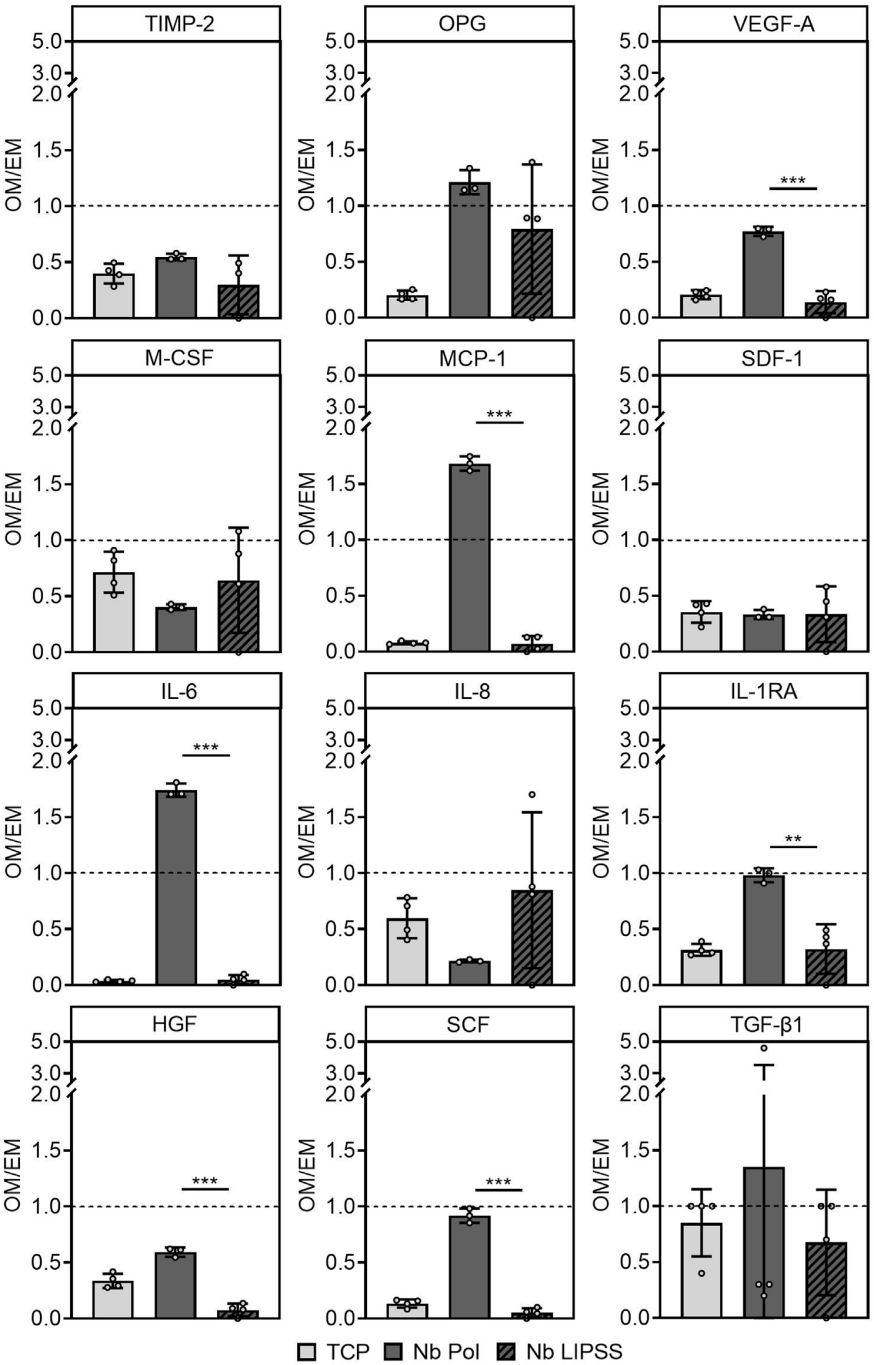
Cytokine and growth factor release of hOBs grown on Ti6Al7Nb. Shown is the release of soluble factors following osteogenic stimulation related to the release of soluble factors without stimulation. (Student’s t-test, n ≥ 3 technical replicates, n = 1 cell donor, * indicates *p* ≤ 0.05, ** indicates *p* ≤ 0.01, *** indicates *p* ≤ 0.001) Abbreviations: Nb POL, polished Ti6Al7Nb disks; Nb LIPPS, UV-LIPSS textured Ti6Al7Nb disks; TCP, tissue culture plastic.

Treatment of Ti6Al4V with UV-LIPSS resulted in a significant increase of metal ion release. In addition, the use of UV-LIPSS on Ti6Al4V significantly decreased the number of adherent cells. Thus, UV-LIPSS treatment of Ti6Al4V seems to trigger unfavorable effects in hOBs. In contrast, metal ion release on Ti6Al7Nb was decreased after UV-LIPSS surface treatment. It was therefore decided to investigate the effect of UV-LIPSS on Ti6Al7Nb further since this alloy seems to have more favorable characteristics. The release of cytokines from cells grown on Ti6Al7Nb surfaces following osteogenic stimulation was quantified (Figure 7). Among the markers for bone resorption and vascularization, only the release of VEGF-A was significantly reduced for hOBs grown on UV-LIPSS surfaces when compared to cells grown on polished surfaces. Amid the cytokines that regulate the recruitment or activation of immune cells, the release of MCP-1 was significantly higher for cells grown on polished Ti6Al7Nb surfaces and its release was found to be more than 1.5-fold increased upon osteogenic stimulation. Among inflammatory cytokines that regulate immune cell response, IL-6 and IL-1RA concentrations were found to be lower after culture on UV-LIPSS surfaces. The release of growth factors HGF and SCF were also found to be significantly lower for cells grown on UV-LIPSS surfaces upon osteogenic stimulation. In summary, osteogenic stimulation affected VEGF-A, MCP-1, IL-6, IL-1RA, HGF and SCF by elevating their release following culture on polished surfaces when compared to UV-LIPSS treated surfaces.

### 3.7 The formation of biofilms was reduced on LIPSS-treated disks

**FIGURE 8:**
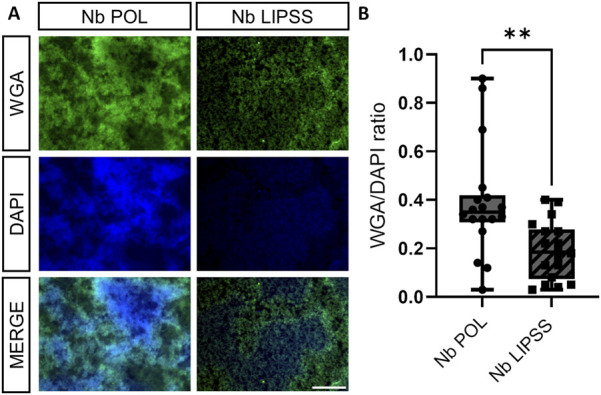
Biofilm formation on polished and LIPSS treated Ti6Al7Nb parts. **(A)** The presence of bacteria-associated and extracellular polysaccharides was visualized via wheat germ agglutinin (WGA) staining. Bacterial and extracellular DNA was visualized using DAPI stain. **(B)** Biofilm formation was quantified by referencing signal intensities of WGA to DAPI signal. (scale bar indicates b = 100 μm, Mann-Whitney Test, n = 18 field of views per surface treatment from n = 6 polished and UV-LIPSS Ti6Al7Nb parts, * indicates *p* ≤ 0.05, ** indicates *p* ≤ 0.01)

To investigate the influence of UV-LIPSS on bacterial attachment and particularly subsequent biofilm formation, the clinically relevant bacterium *S. aureus* (strain INFECT6005/ST20140625) was seeded on polished and LIPSS surfaces of Ti6Al7Nb sample disks. Biofilm formation was decreased on UV-LIPSS treated disks when compared to polished ones (Figure 8A). Quantification of WGA signal intensities confirmed a significant reduction of biofilm formation on UV-LIPSS-treated Ti6Al7Nb disks (Figure 8B). In summary, UV-LIPSS treatment significantly reduced WGA signal intensities pointing towards reduced biofilm formation.

## 4 Discussion

In the course of the present work, the influence of UV-LIPSS on hOB viability, function and the secretion profile was investigated for Ti6Al4V and Ti6Al7Nb.

The alloys Ti6Al4V and Ti6Al7Nb were applied since both are considered biocompatible and long-term experience with these alloys in dental implants and orthopedic endoprostheses is characterized by high success rates with long service lives in patients. The present results show that the release of metal ions was increased for UV-LIPSS surfaces on Ti6Al4V, while the opposite effect was observed for Ti6Al7Nb. The release of metal ions is related to surface processing parameters employed for LIPSS. The energy E required for the surface texturing was found to be different for the two alloys. Ti6Al4V required a lower energy E_p_ input indicated by a difference of 10.6% in energy E_p_ needed to induce UV-LIPSS formation. Both materials are prone to the formation of an oxide layer (TiO_2_). As demonstrated by other researchers, higher energy E_p_ densities on the titanium alloy surface lead to a thicker oxide layer (Dudek et al., 2022). Yet it would need further confirmation by evaluation of the interplay between the employed laser, processing parameters, and microstructure of the oxide layer if differences are present for the two alloys employed and how this might affect metal ion release (Bonse et al., 2002). This information could aid in minimizing metal ion release, since local tissue exposure with metal ions can lead to implant loosening (Schulze et al., 2023). In this regard, it is interesting that intracellular metal ion levels are not affected by the differences in metal ion concentration within the cell culture medium. These observations might be explained by the absence of metal transporter proteins specific for Ti, Al and Nb in eukaryotic cells (Bird, 2015). While investigating cell viability and proliferation, differences were found between the groups tested while acute cytotoxicity was not detected. Initial cell number was significantly lower on UV-LIPSS modified Ti6Al4V surfaces while the proliferation rate was not affected, indicating a decrease in initial cellular adhesion on Ti6Al4V. This observation contrasts with the literature, where LIPSS modifications are generally reported to enhance cellular adhesion (Maalouf et al., 2022). It needs to be noted that the respective studies used IR LIPSS. Laser radiation in the UV range is known to induce LIPSS with smaller periodicities when compared to the IR range. Higher spatial periodicities promote the alignment of cells in the texture direction by contact guidance (Cunha et al., 2015). The differences in cellular adhesion observed in our study might be linked to the submicron-structures formed on the surface. The LIPSS manufactured with UV radiation in this study had a periodicity of 223 nm ≤ λ ≤ 278 nm and a profile height of 45 nm ≤ h_p_ ≤ 98 nm. Another study, which even used a shorter wavelength λ = 193 nm, did not report quantitative evidence for cell adhesion although the authors note that cell adhesion is not influenced by LIPSS modification (Kumari et al., 2015). In the study of Cunah and coworkers, it was reported that albeit cell density is not affected by LIPSS, the amount of focal adhesion points is significantly decreased (Cunha et al., 2015). Yet, further investigations would be required to be able to draw a final conclusion about the diminished adherence of hOBS on UV-LIPSS Ti6Al4V and why similar effects have not been observed for Ti6Al7Nb.

Cells grown on UV-LIPSS modified Ti6Al7Nb surfaces exhibited a significantly lower metabolic activity compared to cells grown on polished Ti6Al7Nb. In this regard, the significantly higher metabolic activity observed for hOBs on polished surfaces might not solely represent enhanced viability. The increase in metabolic activity could also be a sign of a higher oxidative stress that these cells encounter due to the elevated metal ion release found for the polished Ti6Al7Nb surfaces. In this regard, a significantly higher release of SCF was detected when cells were grown on polished surfaces. It is reported that SCF exhibits protection against oxidative stress in OBs (Yang et al., 2014). It is thus likely, that the secretion of SCF increases in response to the elevated metal ion levels observed for polished Ti6Al7Nb in this study. The rate of cell death was not found to be influenced by surface treatment confirming earlier findings regarding UV-LIPSS modified Ti6Al4V (Schweitzer et al., 2020). Osteogenic activity in terms of ALP activity is enhanced for hOBs grown on UV-LIPSS surfaces on both alloys, indicating a positive effect on bone matrix formation. In addition, the release of TIMP-2 and OPG was investigated and found decreased for hOBs grown on LIPSS surfaces. TIMP-2 is an inhibitor of matrix metalloproteinase 2 (MMP-2), an enzyme that is vital for the breakdown of extracellular matrix in bone (Sternlicht and Werb, 2001), while OPG is an inhibitor of the activity of bone-resorbing OC (Udagawa et al., 2021). The reduction of both factors indicates stimulatory effects of bone resorption. This would however need further confirmation by more complex *in vitro* models that include macrophages and OC or *in vivo* experiments.

It was also investigated how the profile of cytokines and growth factor secreted by hOBs changes in response to different surface treatments. Results show that under baseline conditions in EM, MCP-1 secretion by hOBs grown on UV-LIPSS modified Ti6Al7Nb was increased. Yet, when an osteogenic stimulus was applied, hOBs showed distinct cytokine and growth factor secretion. It was found that MCP-1 and IL-6 release by hOBs grown on polished surfaces is significantly higher when compared to UV-LIPSS modified Ti6Al7Nb surfaces. It is reported that OBs secrete MCP-1 in response to osteogenic stimuli or exposure to the inflammatory cytokine IL-6 to induce bone remodeling activity by monocyte recruitment (Raggatt and Partridge, 2010). It is also reported that MCP-1 seems to be involved in aseptic implant loosening (Gu et al., 2012). The increased secretion of MCP-1 by hOBs grown on polished Ti6Al7Nb in combination with elevated levels of IL-6 might hint at an autocrine stimulation feedback loop that targets enhanced monocyte recruitment. In this regard, the receptor activator of nuclear factor κB ligand (RANKL) is essential for survival, proliferation, and differentiation of OC precursor cells (Wada et al., 2006). The release of RANKL increases in response to inflammatory cytokines such as IL-1, while its activity is decreased by the decoy receptor OPG (Proff and Romer, 2009). The tissue availability of IL-1 is modified by its antagonist, IL-1RA (Harrell et al., 2020). We found a significantly higher release of IL-1RA by hOBs grown on polished surfaces when compared to UV-LIPSS, indicating that monocyte recruitment but not differentiation is targeted. A significant higher secretion of HGF was observed for hOBs grown on polished surfaces. The soluble factor HGF is reported to stimulate the growth of hematopoietic cells and also acts as an attractant for monocytes (Galimi et al., 2001). This immunostimulatory signaling is in line with the increased MCP-1 and IL-6 release.

Tissue vascularization is of paramount importance for the formation of vital bone and is mediated by VEGF-A, which is upregulated in response to stimuli such as HGF expression or oxidative stress (Ribatti and d’Amati, 2023). In the current work, VEGF-A release is significantly higher for hOBs grown on polished surfaces when compared to their UV-LIPSS counterparts. Interpretation of the current data in terms of bone formation or resorption *in vivo* should be exercised with caution since proving the respective mechanisms in action would require additional *in vitro* and *in vivo* experiments. Yet, it is evident from the current data set, that inflammatory responses observed in this study seem to be triggered exclusively by polished surfaces, while UV-LIPSS topographies did not alter the cytokine release profile when compared with TCP as the respective control.

In a last step, it was demonstrated that UV-LIPSS treatment leads to a decrease in biofilm formation by the *S. aureus* strain INFECT6005/ST20140625. Albeit this model for biofilm formation is an oversimplification of the clinical reality of polymicrobial biofilms, *S. aureus* is among the most prevalent and thus a relevant pathogen in orthopedic surgery (Pietrocola et al., 2022). It has been hypothesized that the sub-micron structures of LIPSS are smaller than pathogenic bacteria themselves thus effectively reducing available surface area and attachment sites (Siddiquie et al., 2020). In summary, UV-LIPSS treatment of Ti6Al7Nb lead to increased osteogenic activity of hOBs, reduced secretion of pro-inflammatory cytokines and reduced biofilm formation of the clinically relevant pathogen *S. aureus*.

Strength of this work were the preclinical utilization of human primary cells reflecting a real-world arthroplasty clientele, the use of a highly precise and well-characterized state-of-the-art laser technology and the quantification of metal ions released from the novel textures. Limitations of this work might have been the utilization of monocellular *in vitro* models potentially not reflecting the complex cellular interactions at the material interface following implantation. However, this *in vitro* study provides important data for the preclinical testing of commonly used implant materials treated with UV-LIPSS, as this approach identified a very promising surface modification (UV-LIPSS on TiAlVNb). In the next step of the translational process, multicellular and microphysiological *in vitro* models and/or well-established *in vivo* models should be applied to conclusively evaluate the biocompatibility, ingrowth behavior, long-term stability and biofilm formation on UV-LIPSS textured TiAlVNb.

## 5 Conclusion

In this work, we demonstrated the process parameters and the feasibility of UV-LIPSS manufacturing on Ti alloys. The role of LIPSS in shaping the surface topography of implants has generated considerable interest, particularly due to its impact on immunological reactions. We were able to show that the release of metal ions is a function of the alloy and surface modification employed. Depending on the alloy employed, the application of UV-LIPSS did not lead to acute cytotoxicity but influenced cellular adhesion and metabolic activity which both could hint on subchronic effects. Furthermore, UV-LIPSS positively influenced the osteogenic activity of hOBs, while the secretion of pro-inflammatory cytokines was markedly reduced when compared to polished surfaces. In addition, the formation of an *S. aureus* biofilm was significantly reduced on UV-LIPSS surfaces. In conclusion, UV-LIPSS has the potential to positively influence parameters that are vital for proper implant ingrowth and survival and might thus help to increase longevity of orthopedic implants.

## Data Availability

The raw data supporting the conclusions of this article will be made available by the authors, without undue reservation.
